# Dynamic simulation of first pass myocardial perfusion MR with a novel perfusion phantom

**DOI:** 10.1186/1532-429X-13-S1-O43

**Published:** 2011-02-02

**Authors:** Amedeo Chiribiri, Andreas Schuster, Masaki Ishida, Gilion Hautvast, Niloufar Z Nooralipour, Matthias Paul, Shazia Hussain, Philip Batchelor, Marcel Breeuwer, Tobias Schaeffter, Eike Nagel

**Affiliations:** 1 King’s College London, Wellcome Trust – EPSRC Centre of Excellence in Medical Engineering, London, UK; 2 Philips Healthcare, Imaging Systems - MR, Best, Netherlands; 3 King’s College London, NIHR Biomedical Research Centre, London, UK; 4 King’s College London, Division of Imaging Sciences, London, UK; 5 Philips Healthcare, PCCI Clinical Science & Advanced Development and Eindhoven University, Eindhoven, Netherlands

## Objective

To describe a novel hardware MR-perfusion phantom to model first-pass of a bolus of contrast in the cardiac cavities and large thoracic vessels (arterial input function, AIF) and in the myocardium, allowing for the acquisition of dynamic first-pass MR-perfusion data and for a precise control of cardiac output and myocardial perfusion for true validation of perfusion quantification.

## Background

Development of quantitative MR-perfusion would benefit from the availability of a MR-compatible harware phantom allowing for a realistic simulation of the AIF and of the myocardial signal intensity curves. So far, mathematical phantoms or static samples with different T1 values have been described and a tool for development and true validation of MR-perfusion is lacking.

## Methods

Our MR-compatible phantom resembles the anatomy of the heart and of the thoracic vessels of a 60 kg subject. Water flow (2-4 l/min) is driven into the system by a pump located outside the MR room. Gadolinium injections (Gadovist, gadobutrol, Bayer Schering, Germany) are performed in the vena cava by a power-injector (Medrad Solaris, Germany). Progressive dilution of the Gadolinium bolus across the chambers generates the AIF. A fraction of the cardiac output perfuses two myocardial compartments (made by arrays of parallel tubes) where perfusion can be controlled precisely and independently. Outside the MR room, a control unit allows for precise measurement and control of cardiac output and myocardial perfusion. The phantom was tested in a 3T-scanner (Philips Achieva, Netherlands). Acquisition of the perfusion sequence was repeated multiple times with a k-t SENSE sequence (flip-angle=20°; TR2.2 ms; TE1.1 ms; k-t factor 5, 11 training profiles) using similar flow conditions to demonstrate the reproducibility of the measurements. Different flow conditions were used to assess the response of the system to different myocardial perfusion rates. Quantification was performed by Fermi deconvolution.

## Results

**Figure 1:**
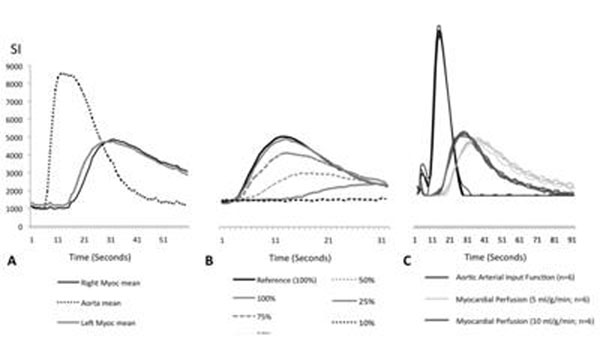
A. Arterial function in the aorta and signal-intensity (SI) in the right and left myocardial compartments (myocardial perfusion rate 10ml/g/min; cardiac output 3 l/min). B Dynamic response of the system to different myocardial perfusion rates (10, 7.5, 5, 2.5, 1 ml/g/min, corresponding to 100%, 75%, 50%, 25% and 10% of the reference). C. Reproducibility of the measurements for constant values of myocardial perfusion rates (5 and 10 ml/g/min) and AIF (with a cardiac output of 4 l/min).

The system produced realistic dynamic first-pass perfusion data (Figure 1A) and reacted very sensitively on alterations of perfusion-rate (Figure 1B). The phantom generated highly reproducible signal-intensity curves for similar flow conditions (Figure 1C and Table 1).

**Table 1:** Experimental conditions and calculated myocardial perfusion rates (Fermi deconvolution) for the dynamic test and the reproducibility test represented in Figure 2 B and C respectively.

	Dynamic response test	Reproduciblity test (n=6)
Cardiac output (l/min)	3 l/min	4 l/min
Right myocardial perfusion	10-7.5-5-2.5-1 ml/g/min	5 ml/g/min
Fermi deconvolution results - Right myocardial perfusion	9.8, 6.4, 4.0, 2.0, 0.9 ml/g/min, respectively	5.5±0.2 ml/g/min
Left myocardial perfusion	10 ml/g/min	10 ml/g/min
Fermi deconvolution results - Left myocardial perfusion	10.8 ml/g/min	9.5±0.2 ml/g/min
Injected Gadobutrol volume	0.6 ml	0.6 ml
Speed of Gd injection (ml/s) followed by 20 ml of saline	4 ml/s	4 ml/s

## Conclusions

This novel hardware perfusion phantom allows reliable, reproducible and efficient simulation of myocardial perfusion. The availability of a direct comparison between the image data and reference values of flow and perfusion will allow for rapid development and validation of accurate quantification methods.

